# Sequential immunotherapy in a patient with primary refractory Hodgkin lymphoma and novel mutations

**DOI:** 10.18632/oncotarget.25037

**Published:** 2018-04-17

**Authors:** Richard Greil, Lisa Pleyer, Bettina Jansko, Carmen Feierabend, Lukas Rettenbacher, Olga Stiefel, Christoph Rass, Patrick Morre, Daniel Neureiter, Sigrun Greil-Ressler

**Affiliations:** ^1^ IIIrd Medical Department with Hematology, Medical Oncology, Hemostaseology, Infectious Disease and Rheumatology, Oncologic Center, Paracelsus Medical University, A-5020 Salzburg, Austria; ^2^ Salzburg Cancer Research Institute, A-5020 Salzburg, Austria; ^3^ Cancer Cluster Salzburg, A-5020 Salzburg, Austria; ^4^ Department of Nuclear Medicine, Paracelsus Medical University, A-5020 Salzburg, Austria; ^5^ Ordensklinikum Linz, A-4010 Linz, Austria; ^6^ Institute of Pathology, Paracelsus Medical University, A-5020 Salzburg, Austria

**Keywords:** immunotherapy, molecular targets, gene mutations, lymphoma

## Abstract

Primary resistant Hodgkin lymphoma is an aggressive disease with few treatment options and short survival. Neoplastic cells of classical Hodgkin lymphoma are heavily dependent on microenvironmental stimuli, regularly express PD-L1, and a relevant proportion of relapsed patients is sensitive to blocking of the PD1/PD-L1 axis. However, response duration is limited and further treatment options are unknown but urgently needed.

We report a case of a patient without relevant response to five subsequent chemotherapy regimens who immediately and dramatically responded to an anti-PD1 mab. During the following two years she responded to the anti-CTLA-4 mab ipilimumab, the Jak2 inhibitor ruxolitinib, and a combination of lenalidomide plus cyclophosphamide given in subsequent relapses. A thorough genomic analysis demonstrated seven genomic alterations with six of them not previously described in this disease (i.e. BRIP1 G212fs*62, KRAS L19F, KDM5A R1239W, MYC A59T, ARIDA1A E1683fs*15 and TP53 277Y). Three alterations were considered actionable and one of them drugable. The number of mutations increased over time and the BRIP1 mutation was found to be a germline mutation.

## INTRODUCTION

Risk-adapated designs of conventional chemotherapy and radiotherapy have led to remarkable high cure rates in Hodgkin lymphoma with 94.8% 5 year failure-free survival in early stages with as few as two cycles of ABVD and involved field irradiation [1] and 91.4% 3year PFS rates with 6 cycles of BEACOPP escalated in advanced stages [2]. However, 10 year failure rates may be still as high as 28% for BEACOPP escalated and 36% for ABVD [3]. Prognosis is dismal in patients with relapse and even worse in those with primary resistant disease who require high-dose chemotherapy with autologous stem cell transplant or allogeneic transplant in order to achieve long-term remission or cure, although the proportion of patients rescued by these approaches is disappointingly low [4, 5]. These patients definitely require new drugs and treatment approaches. Concerning the fact that 20–44% of all patients are older than 60 years [6–8] and cannot stand intensive chemotherapy or even 4 cycles of ABVD and fare poorly under such treatment [9] further emphasizes the need for new strategies.

Targeted therapies with high tolerability and modes of action different from chemotherapy are therefore needed in a substantial proportion of patients as rescue therapy and may substitute for chemo- or radiotherapy with their untoward long-term side effects in the future. However, adequate molecular targets for such strategies are few in classical Hodgkin lymphoma with anti-CD20 mabs failing in this setting [2]. The armed anti-CD30 mab Brentuximab vedotin has proven efficacy in patients in relapse [10] but with only few patients gaining long-term benefit [11]. Nevertheless, the drug is moving forward into first line strategies.

Hodgkin lymphoma is characterized by a rich spectrum of immunological and inflammatory cells surrounding and feeding the neoplastic cells and production of a concert of cytokines which suppress lymphoma-directed immunity [12]. PD-L1 is genomically altered and overexpressed in a relevant proportion [13, 14] and involved in exhaustion and suppression of antitumoral immunity. Anti-PD1 mabs have shown to be successful in relapsed and some refractory patients [15–17] and attract high interest for further development of immunological strategies [18]. However, efficient treatment after failure to anti-PD1 mabs is highly required and new treatment options and novel targets of personalized therapy are needed. We report a case of a primary chemorefractory patient with classical Hodgkin lymphoma sensitive to anti-PD1 mab and to three further immunological treatments in subsequent relapses. In addition, analysis by comprehensive next generation sequencing (NGS) revealed 6 novel mutations previously not described in Hodgkin lymphoma with one of them occurring in germline. The results obtained may be useful for further development of personalized medicine in this disease.

## RESULTS

In September 2014, a then 29 year old woman presented to us with a 76 months history of primarily resistant Hodgkin lymphoma, first diagnosed in May 2008 as classical Hodgkin lymphoma, nodular sclerosis subtype 1, stage IIB with risk factors. The patient received 8 cycles of BEACOPP escalated with some clinical response, but the first post-treatment ^18^FDG PET/CT showed new lesions (November 2008). The responsible physicians were not sure as to whether consider primary resistance or infection and in agreement with the patient suggested a watch and wait policy. During the next 59 months the disease continuously progressed with the occurrence of Pel-Epstein fever and novel nodes confirmed by ^18^FDG PET/CT. In July 2013, mediastinal bulky disease together with signs of heavy pruritus had developed and the patient received two cycles of DHAP (Dexamethason, Cisplatin, high-dose cytosine-arabinoside) without clinical or radiological response. She was switched to brentuximab-vedotin but within the first cycle rapid and life-threatening progression of the mediastinal bulk with obstruction of the bronchus and pulmonary infiltration developed and the responsible physicians decided for emergency pneumonectomy. Further two cycles of brentuximab-vedotin were without success but the patient responded with a CR to the subsequent R-GemOx (Rituximab, Gemcitabine, Oxaliplatin) regimen, unfortunately with a simultaneous failure in stem cell mobilization. Within three further months (i.e. July 2014) she again was clinically and radiologically progressive. She received DexaBEAM (Dexamethasone, BCNU, etoposide, cytarabin, melphalan) without remission and refused the allogeneic stem cell transplant offered to her.

**Figure 1:**
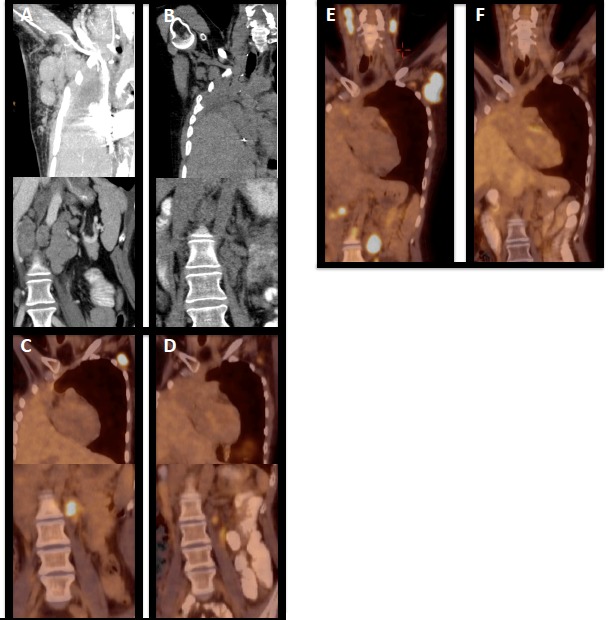
Course of disease under immunologic treatment (**A**) CT scan showing massively enlarged axillary and subdiaphragmal lymph nodes prior to the start of nivolumab, (**B**) CT control two months after start of nivolumab showing massive regression of nodes in both regions, (**C**) ^18^FDG PET/CT in progression during nivolumab and prior to start of ipilimumab (**D**) treatment response during ipilimumab (**E**) ^18^FDG PET/CT cervical, axillary , and subdiaphragmal lymph node progression after ipilimumab and prior to start of ruxolitinib (**F**) ^18^FDG PET showing ruxolitinib-induced remission.

When she presented to us, the patient suffered from severe B symptoms, showed massive nodes, had a highly inflammatory lab and underwent a combination of pixantrone, paclitaxel and G-CSF. She developed sepsis with opportunistic infection from which she slowly and only partially recovered. In February 2015, then 83 months after the primary diagnosis, the patient was severely ill with tachycardia, dyspnea, multiple small nodular pulmonary infiltrations, a 5 cm pericardial infiltration, large mediastinal, hilar and subdiaphragmatic nodes as well as new liver lesions (stage IVEB) (Figure 1A). Her LDH was 492 U/l (normal range 135-225 U/L), CRP 23.7 mg/dl (normal range < 0.6 mg/dl), the temperature was 37° C without definite signs of infections, her blood pressure had dropped to 87/58 mmHg, and her heart beat rate was 126/min.

Nivolumab at a dose of 3 mg/kg was started and the patient improved immediately even during infusion (vanishing of pruritus, cardiovascular improvement). She achieved a PR as documented by CT scan as of May 2015 (Figure 1B) and was kept on the drug with excellent tolerability and without relevant side effects. In October 2015 she developed pruritus and the control ^18^FDG PET/CT scan in November 2015 showed relapse in cervical, retrolaryngeal, subpectoral and axillary nodes (Figure 1C). Due to the previous pneumonectomy radiotherapy was withheld, nivolumab was stopped and the patient was switched to ipilimumab at a dose of 3 mg/kg beginning in November 2015. The patient again achieved a clinical response and a PR in ^18^FDG PET/CT scan (Figure 1D). However, in March 2016 elevations of liver enzymes, i.e. glutamat-oxalacetat-transaminase (NCCT grade 4), alanin-aminotransferase (NCCT grade 4), gamma glutamyltransferase (NCCT grade 3), alkaline phosphatase (NCCT grade 3), and bilirubin (NCCT grade 2) were observed although the liver synthesis capacity remained unchanged. The ^18^FDG PET/CT in April 2016 showed a mixed response with a significant remission in axillary lymph nodes on the left side, but with some new neoplastic lesions in the right axilla. Ipilimumab was stopped due to hepatotoxicity and steroids implemented. Her liver values recovered slowly and completely, but unfortunately she further progressed from Hodgkin lymphoma with rapidly growing and symptomatic nodes in June 2016 (Figure 1E), which was histologically confirmed. She was switched to ruxolitinib at a starting dose of 20mg daily (10 mg bid) and then 50 mg daily (25 mg bid)) and again within days showed decrease in node size, pruritus and B symptoms and she entered remission in ^18^FDG PET/CT scan in September 2016 (Figure 1F). She relapsed in November 2016 underwent radiotherapy of skeletal disease and due to enlarged lymph nodes in February 2017 was switched to lenalidomide 20 mg q21d and cyclophosphamide 50 mg/po q28d and again showed immediate shrinkage of lymph nodes. As of October 2017 the patient is very well without any B symptoms and without restrictions in her daily life. Treatment is associated with a complete vanishing of palpable nodes, which recur when treatment has to be stopped for reasons of drug-induced cytopenia, but nodes rapidly disappear within days when treatment is started again.

**Figure 2:**
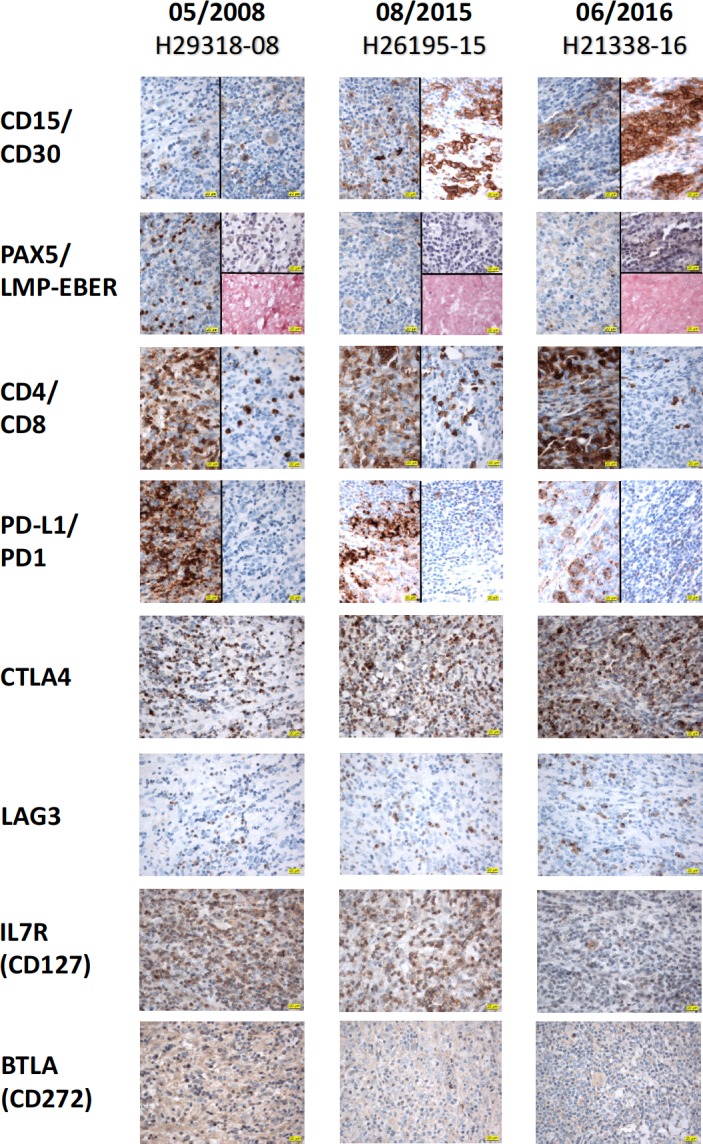
Immunohistochemistry Three lymph node biopsies were taken in May 2008 (initial diagnosis, column 1), in progression after 8 cycles of nivolumab (August 2015, column 2), and after 12 cycles of nivolumab and treatment with 7 cycles of ipilimumab (June 2016, column 3). The obtained specimens were comprehensively analysed for diagnostic (CD15, CD30, PAX5, LMP, In-situ-Hybridization for EBER, CD4 and CD8) and possible therapeutic (PD-L1, PD1, CTLA4, LAG3, IL7R (CD127) and BTLA (CD272)) purposes with immunohistochemistry. First, the histomorphological Reed Sternberg and Hodgkin cells could be detected with CD15 and CD30 (lane a) and PAX5 (weak, lane b). An EBV-association was excluded by negative staining for LMP1 and *in-situ*-hybridization for EBER (lane b). Second, PD-L1 expression on Hodgkin cells decreased from the initial biopsy with the highest protein expression pattern in comparison to biopsies taken in relapse after treatment with nivolumab and ipilimumab (lane d). Interestingly, the PD1 expression (lane d) in the surrounding Hodgkin-associated lymphocytic bystander population with predominant CD4-positive phenotype (lane c) was low. Third, intensive analysis of the immunological checkpoint proteins CTLA4, LAG3, IL7R (CD127) and BTLA (CD272) revealed an increase of CTLA4 and a decrease of IL7R and BTLA by continuously low expression pattern for LAG3 throughout the different time periods (lanes e to h). (Note that the first antigen designated at the left side of each lane always depicts the left part of the microphotograph, whereas the antigen given after the slash describes the relevant right part of the photo, respectively. Magnification of 1 × 400 for all immunohistochemical pictures).

For confirmation of histology as well as for the potential identification of immunological and molecular targets valuable for future treatment options, lymph nodes were excised when the patient was in progression after the 8th application of nivolumab (7th line of therapy; August 2015), and again in progression after ipilimumab (8th line of therapy; June 2016). These specimens as well as material from the initial lymph node biopsy taken from the chemonaive patient in May 2008 were investigated by an experienced hematopathologist and stained for markers specific for Hodgkin lymphoma as well for checkpoint molecules potentially involved in the regulation of anti-lymphoma immunity (Figure 2). Reed Sternberg and Hodgkin cells coexpressed CD15 and CD30 (Figure 2, lane a), CD20 and nuclear MUM1; the number of Ki67 positive cells was low (results not shown). Aberrant weak expression of PAX5 was seen (Figure 2 lane b). EBV-association was excluded by negative staining for LMP1 and *in situ* hybridization for EBER (Figure 2, lane b). PD-L1 expression on Hodgkin cells was high in the initial biopsy, but was significantly decreased in the nodes taken in relapse after treatment with nivolumab and ipilimumab, respectively (lane d). The surrounding Hodgkin-associated lymphocytic bystander population showed a predominant CD4-positive phenotype (lane c) with really low expression level of PD1. Furthermore, comprehensive analysis of the immunological checkpoint proteins CTLA4, LAG3, IL7R (CD127) and BTLA (CD272) showed an increase of CTLA4 and a decrease of IL7R and BTLA at the different time points, whereas the expression of LAG3 was continuously low (lanes e,f,g,h).

**Figure 3:**
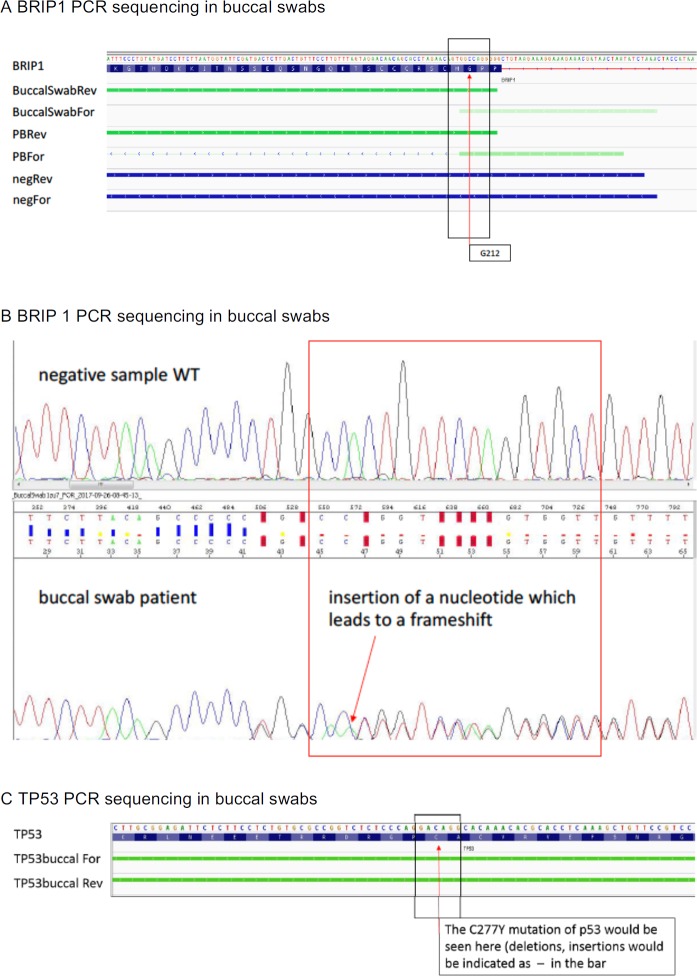
PCR sequencing of germline DNA (**A**) IGV Alignment BRIP1 G212fs*62: BRIP1 G212fs*62 mutation was detected in buccal swab DNA and in peripheral blood DNA of the patient. The forward and reverse sequence alignment stops at G212 due to the frameshift in the mutated sample. Negative control sample aligns perfectly to the reference sequence. BRIP1: reference sequence of BRIP NM_032043; BuccalSwabRev: DNA from buccal swab of the patient (reverse strand); BuccalSwabFor: DNA from buccal swab of the patient (forward strand); PBRev: DNA from peripheral blood (PB) of the patient (reverse strand); PBFor: DNA from PB of patient (forward strand), negRev: negative control DNA from PB (reverse strand); negFor: negative control DNA from PB (forward strand). (**B**) BRIP1 nucleotide insertion leads to a frameshift mutation. (**C**) IGV Alignment TP53 Exon 8: TP53 C277Y mutation could not be detected in buccal swab DNA. Alignments are identical to the reference sequence. TP53: reference sequence (NM_001126114), TP53 buccal For: DNA from buccal swab of patient. TP53buccal Rev: DNA from buccal swab of patient.

In addition, material from all the three lymph node samples as well as DNA from peripheral blood obtained in progression after ruxolitinib in May 2017 were sent out for analysis by NGS to Foundation One (Tables 1 and 2). Over the course of the disease, seven genes were found to be altered (Table 2), with mutations in BRIP1 G212fs*62, KRAS L19F, MYC A59T, ARIDA1A E1683fs*15, KDM5A R1239W and TP53 277Y to the best of our knowledge never reported before in Hodgkin lymphoma. Over time, the mutational architecture became more complex. Two mutations were present at the time of initial diagnosis. 97 months later and after 6 lines of chemotherapy and two lines of immunotherapy five mutations were observed with only two of them recognized initially (Table 2). Analysis of DNA of peripheral blood carried out in progression after ruxolitinib suggested germline mutation for BRIP1 and this was confirmed by DNA collected from peripheral blood and buccal swabs (Tables 1, 2 and Figure 3A, 3B). In addition, the liquid biopsy demonstrated the presence of a TP53 C227Y mutation in the circulating free DNA which was not caused by a germline mutation (Figure 3C).

**Table 1:** Results from NGS testing

Genomic finding detected	Previously described in Hodgkin lymphoma^1^	FDA-approved therapies in patients’ tumor type	FDA-approved therapies in another tumor type	Potential clinical trials
BRIP1 G212fs*62^2^	Not found in any of 9 Hodgkin lymphoma cases in the Cosmic database (Dec 2016); Not studied according to Pubmed in Hodgkin lymphoma (August 2017)	None	Olaparib^3^	NCT00576654
KDM5A R1239W	No reports in Hodgkin lymphoma (Pubmed, August 2017)	None	None	None
KRAS L19F	Not identified in 48 Hodgkin lymphoma samples analyzed in COSMIC database (Dec 2016), no significant reports in Pubmed /August 2017)	None	Trametinib, Cobimetinib	NCT01742988 NCT01991938
MYC A59T	No MYC mutation found in 9 cases of Hodgkin lymphoma analyzed in COSMIC database (August 2017)	None	None	NCT02431260 NCT01943851 NCT01949883
ARIDA1A E1683fs*15 ^4^	No reports	None	None	None
B2M M1R	Inactivating mutations described in classical Hodgkin lymphoma	None	None	None
TP53 C277Y	No descriptions in Hodgkin lymphoma Pubmed, August 2017	None	None	None

^1^Data is given according to reports from FoundationOne. Descriptions are given according to COSMIC database.

^2^BRIP1 G212fs*62 equivalent to FANCJ (Fanconi Anemia complementation group J). The gene is considered important for DNA repair and maintenance of chromosomal stability. The observed alteration disrupts the BRCA1 binding site and is predicted to be inactivating.

^3^A patient with serous ovarian cancer harboring a BRIP1 mutation exhibited a long-lasting response to olaparib [47].

^4^ARIDA1A is supposed to be a tumor suppressor and part of the chromatin remodeling complex. Mutations are reported to occur in 2% of hematopoietic and lymphoid malignancies (COSMIC 2016). ARIDA 1 expression has been associated with dexamethasone resistance [59].

Variants of unknown significance: CAD P1465L, DDX3XA541V, FLT1 R1257C, GNA13 E138QK121fs*4, HDAC4 Q154H, KDM4C S436G, MAFB H166R, PCLo R3605Q, PDGFRA T200S, RELN P672L, SDHA G3V, SETB1 K1341E, TSC1 N762S, Tumor mutation burden unknown, XPO1 R231I, ZNF703 A401–H402insPTH LGGSSCSTCSA.

**Table 2:** Genomic alterations in lymph node biopsies, liquid biopsy and germline

Time point after x lines of therapy	Most recent therapy	Subsequent therapy	Tissue	Genomic Findings
Chemo-naive, May 2008	None	BEACOPP escalated	pretracheal lymph node^1^	(1)BRIP1 G212fs*62 (2)MYC A59T
August 2015, during 7th line	8 cycles of nivolumab	4 cycles of nivolumab 7 cycles of iplimumab	lymph node cervical/supraclavicular^1^	(1)BRIP1 G212fs*62 (2)KDM5A R1239W
June, 2016 after 8th line	8 lines of therapy including nivolumab and ipilimumab	ruxolitinib	lymph node accessorius region left^2^	(1)BRIP1 G212fs*62 (2) MYC A59T (3)KRAS L19F (4)ARID1A E1683fs*15 (5)B2M M1R
July 2017 after 9th line	ruxolitinib	cyclophos-phamide/lenalidomide	peripheral blood^2^	(1)BRIP1 G212fs*62 (2)TP53 C277Y
July and October 2017	cyclophos-phamide/ lenalidomide	treatment ongoing	buccal swap^3^	1)BRIP1 G212fs*62

^1^Results according to Foundation One which interrogates 315 genes as well as introns of 28 genes involved in rearrangements.

^2^ Results according to Foundation One Hem test which analyzes 406 genes as well as selected introns of 31 genes involved in gene rearrangements, and performs RNA sequencing of 265 genes.

^3^ performed in house (for details see Material and Methods section and Figure 3). With the only exception of B2MG, all genes are tested in both test systems.

With the exception of the B2MG gene, all genes analyzed were included in both panels of Foundation One.

## DISCUSSION

### The course of disease for this patient is remarkable for several reasons

First, relapsed/refractory Hodgkin lymphoma provides a therapeutic dilemma. High dose chemotherapy with autologous stem cell transplant is considered standard of care for relapsed patients with improved PFS but questionable OS benefit over conventional rescue chemotherapy regimens [5, 19, 20]. Shortened time between first diagnosis and relapse (<12 months), anemia, B symptoms, bulk at initial presentation, involvement of extranodal sites [5] as well as positive ^18^FDG PET [21], >3 salvage regimens and chemoresistance predict high risk and unfavorable course. Even with autotransplant, only 41% of patients with a high risk score were progression free after 4 years in a recent study [22]. In groups of purely primary refractory patients, prognosis was even worse with 5 year OS rates of 36% [23] to 48% [24] and in case of simultaneously present poor risk factors 28% [21]. Due to the extremely poor prognosis of patients put on conventional chemotherapy (e.g. OS of 3 to 16 months [25, 26]), immediate proceeding to high dose chemotherapy and autotransplant probably with double transplantation in high risk patients is recommended in these patients [4, 27].

In this case, the initially responsible physicians and the patient herself could not reach consensus on the early implementation of autotransplant, and at the time they consented, stem cells could not be collected any more, supporting the necessity for early transplant. Out of six lines of therapy including brentuximab vedotin, the patient responded to only one and for a period of only 3 months. Given the simultaneous presence of a very high risk score the survival time of the patient without any efficient therapy is remarkable.

Second, the patient was immediately life-threatened by her disease in February 2015 and she received nivolumab 3 months after the first presentation of efficacy data of the drug at the American Society of Hematology, December 2014 [15], although the drug was not commercially available at that time in Europe. The effect was extremely fast, despite the fact that the patient presented with nearly all risk factors currently defined for this disease. Checkpoint inhibitors cause significant benefit in patients in relapse after autologous stem cell transplant ± brentuximab treatment or after brentuximab-treatment in case of ineligibility for transplant, with RR/CR rates between 87%/17% [15], 67%/9% [16] for nivolumab and 64%/16% for pembrolizumab [17] and median durations of all responses of 7.8 months for nivolumab [16] and 70% longer than 6 months for pembrolizumab [17]. However, treatment recommendations for patients relapsing after anti-PD1 and brentuximab vedotin as in this case do not exist to our knowledge and options are few.

Sequential administration of different checkpoint inhibitors have been shown efficient in melanoma, with the sequence of nivolumab followed by ipilimumab proving more efficient than the inverse sequence [28]. However, only few cases of heavily pretreated patients with Hodgkin lymphoma (all after allo-transplant) have been reported in the literature, with 2/14 achieving a CR after ipilimumab [29]. We are not aware of a report on the sequential use of nivolumab and ipilimumab in this disease and find it notable that the patient developed a remission after ipilimumab again. This may be important for the design of future trials. Combined inhibition of PD1 or PD-L1- and CTLA4-mediated suppression of anti-lymphoma immunity seems supported by the finding that CTLA4 significantly increased during treatment with nivolumab and ipilmumab and remained so after the stop of treatment with this drug (Figure 2, lane e). LAG 3 [30] and BTLA4 [31] have been involved in the immunosuppression in Hodgkin lymphoma and synergistic effects in the restitution of the exhausted immune response between anti-PD1 and anti-LAG3 have been shown in several tumor systems [32, 33]. However, LAG 3 expression in Hodgkin lymphoma has predominantly been observed in EBV+ cases [30]. This case was EBV- and in fact the initial expression of LAG3 and BTLA was very weak and weak respectively (Figure 2, lanes f and h). The former remained low and the latter even decreased further during immunotherapy. These results would not favor targeting these molecules together with PD1 in combination strategies at least in a case similar to ours.

Third PD-1L was strongly expressed on Sternberg Reed cells in the present case (Figure 2, lane d), but PD-L1 was neither amplified, translocated nor mutated although copy number alterations, amplifications [13, 14] and translocations of MHC class II transactivator CII TA with subsequent activation of PD-L1 and PD-L2 [34] are frequent in this lymphoma and associated with advanced stage and inferior outcome of first line therapy [14, 35]. EBV-induced expression of LMP1 has also been shown to activate PD-L1 expression but this case was EBV and LMP1 negative [36]. However, proinflammatory cytokines released from the micromilieu like TNFa [37] and IFNg [38] may be responsible for upregulation of PD-L1 in this case.

Fourth, JAK2 gene amplification cooccurrs with PD-L1 amplification in Hodgkin lymphoma [13] and the amplified JAK2 gene dosage further increases PD-L1 expression. However, no genomic alterations of the JAK1, JAK2, and JAK3 genes were detected in our case. This is important since very recently JAK2 gene mutations have been involved in resistance against PD1 blockade [39].

JAK 2 inhibition has proven efficient *in vitro* and in xenotransplant experiments [40] and JAK2inhibition therefore is a reasonable approach in Hodgkin lymphoma. Inhibition of JAK2 may counteract prosurvival pathways in this disease [40], downregulate PD-L1 expression and enhance immunogenicity [13, 38]. In fact, three of seven patients who had relapsed after conventional chemotherapy (not including checkpoint inhibitors) responded to the drug in a small trial [41]. The fact that the patient achieved an ^18^FDG PET/CT-confirmed remission with a very rapid improvement in clinical symptoms is encouraging.

Fifth, even in relapse after ruxolitinib, the patient again responded to an immunomodulatory regimen consisting of lenalidomide and cyclophosphamide previously shown effective in resistant or relapsed Hodgkin lymphoma [42]. The fact that four immunomodulatory regimens applied in sequence to a life-threatened patient without relevant response or response duration to six previous chemotherapy regimens underlines the therapeutic potential of this approach.

In addition, the occurrence of a mutation within the B2MG gene as in this case is usually considered to interrupt the structure and function of the MHC I complex and antigen presentation thus contributing to immune escape. It has been described as the most frequent mutation of Sternberg Reed cells [43]. Decrease or absence of B2Mg/MHC I has a negative prognostic impact independent of PD-L1/PD-L2 amplification [35]. This contrasts with the high efficacy of anti-PD1 and anti-CTLA4 strategies and may point to effector mechanisms of targeting PD1 and CTLA4 apart from restitution of CD8+ effector cells from exhaustion, like e.g. elimination of Treg cells [44] and evolution and diversification of the TCR repertoire [21] by anti-CTLA4 abs and inhibition of generation, maintenance and function of T reg cells [45] and expansion of NK cells by anti-PD1 targeting [46].

Sixth, the extensive molecular analysis revealed six mutations which to the best of our knowledge have not previously been described in Hodgkin lymphoma. Three of these mutations were considered actionable (Table 1) and at least the BRIP1 G212fs*62 mutation might be directly targeted by PARP inhibitors like olaparib [47]. The BRIP1 mutation was clearly a germline mutation as demonstrated by its presence in all tumor samples analyzed and its presence in DNA extracted from peripheral blood and buccal swabs (Figure 3). BRIP1 germline mutations have recently been shown to be associated with an increased risk in epithelial ovarian cancer and some high grade ovarian serous disease. A truncating BRIP1 mutation has been identified in a small proportion of women suffering from epithelial ovarian cancer in Iceland [48]. In a recent investigation of 3,236 patients with epithelial ovarian cancer and 2,000 control persons deleterious and missense mutations were associated with an 11.22 times increased risk for epithelial ovarian cancer [49] leading to an estimate of a weak to moderate risk of 3.41 times in mutation carriers as compared to the general population. Similar findings were obtained in an analysis from patients with ovarian cancer recruited to clinical trials [50]. In addition, BRIP1 germline mutations have been identified in breast cancer patients [51, 52], and in prostate cancer patients with at least one additional cancer [53]. The family history of the patient showed only one case of breast cancer in her grandmother occurring at the age of 83, and a benign neuroendocrine tumor of the intestine in her father, but no evidence of an increased familial cancer risk in general and for cancer types associated with BRIP1 mutations until now. To the best of our knowledge, this is the first report of a BRIP1 germline mutation in Hodgkin lymphoma. The frequency of this mutation should be analyzed in a larger number of patients. If confirmed, it might have impact on potential novel treatment strategies as well as follow-up investigations in cancer survivorship programs for this disease.

Finally, analysis of cf DNA in blood drawn after ruxolitinib and prior to the most recent treatment regimen also showed the presence of a TP53 C277Y mutation which was not caused by a genomic germline alteration (Figure 3C). Despite the very low number of Reed Sternberg and Hodgkin cells present in Hodgkin lymphoma genomic alterations have been demonstrated in cf DNA even in early stages of disease and these aberrations correlated well with results from lymph node biopsies [54]. Clonal evolution during immunotherapy thus is the most likely interpretation of this finding. Such an evolution is certainly intriguing. There may be a correlation between the mere number of mutations per megabase defined as mutational burden and response to checkpoint inhibitors though this correlation may significantly differ in distinct cancer entities [55]. However, mutational burden is difficult to define in tumors with such a low tumor cell content as is the case in Hodgkin lymphoma and in fact could not be evaluated in our case. The interrelation between defined genomic alterations and their development over time and specific immune reactions against potentially resulting neoantigens and even more the influence of certain immunomodulatory strategies on this interaction in individual cases is extremely complex. The understanding of these processes is in its infancies and requires extensive workup with sophisticated techniques. [56]. Such investigation was outside of the scope of this study which however supports the necessity for systematic work in this regard.

Taken together, this case shows the remarkable effects of sequentially applied immunomodulatory drugs in Hodgkin lymphoma and offers possible solutions for desperately ill patients with this disease but may also be useful for considering novel treatment designs in clinical trials even in earlier stages of disease.

## CONCLUSIONS

Immunotherapy has a high potential in chemoresistant Hodgkin lymphoma with no cross-resistance to different immunomodulators. Response with the drugs chosen after failure of anti-PD1 mab has not been described and the results may be important for the design of novel even chemo-free regimens. Novel actionable and drugable targets are reported.

## METHODS

### Immunohistochemistry (IHC)

IHC was done on the lymph nodes excised at the inital diagnosis of the Hodgkin lymphoma (05/2008) as well as in relapses after immunotherapy (early 08/2015 and late 06/2016). IHC was routinely done on FFPE tissue. In brief, 4 µm sections were mounted on glass slides, deparaffinized with graded alcohols, and stained using the following primary abs: anti-CD-4 (rabbit monoclonal (rm), clone SP35, Ventana, ready-to-use (rtu)), anti-CD-8 (rm, SP57, Ventana, rtu), anti-CD-15 (mouse monoclonal (mm), MMA, Ventana, rtu), anti-CD-20 (mm, L26, Ventana, rtu), anti-CD-30 (mm, Ber-H2, Ventana, rtu), anti-PAX5 (rm, SP34, Ventana, rtu), anti-Ki67 (rm, 30-9, Ventana, rtu), anti-Mum1 (rm, MRQ-43, Cell Marque, rtu), anti-PD-1 (mm, Nat105, Ventana, rtu) and anti-PD-L1 (rm, 28-8, Abcam, dilution 1:300) as well as anti-LMP (mm, CS.1-4, DAKO, rtu). In addition, the following anti-checkpoints-antibodies were immunohistochemically established: anti-CTLA4 (mm, clone BNI3, Abcam, 1:50), anti-LAG3 (rm, EPR20261, Abcam, 1:500), anti-IL7R alpha (CD127) (rabbit polyclonal (rp), clone not stated, Abcam, 1:200) and anti-BTLA (CD272) (rp, clone not stated, Abcam, 1:800). All immunohistochemical stainings were performed on a Benchmark Ultra (Ventana) platform with the OptiView DAB IHC detection kit for PD-1, PD-L1 and CTLA4 antibodies or UltraView Universal DAB detection kit (both kits Ventana) for all other antibodies. Finally, the chromogenic *in situ* hybridization for EBER (Ventana) was applied according the manufactory instructions using the same platform.

### Genomic analysis

Tissue sample from the three lymph nodes excised at different time points and DNA extracted from peripheral blood were sent to FoundationOne in Boston, MA, USA.

### Sequencing of germline DNA

DNA from peripheral blood and buccal swab was isolated according to the technical manual Maxwell 16 Buccal Swab LEV DNA Purification Kit.

### BRIP1 G212fs*62 mutation

Primers for Exon 7 and PCR conditions were chosen according to Lewis *et al.*, 2005 [57]. The following primer sequences were used: BRIP Exon7 For 5′ -> 3′: TTCCATGTGAGGTTTGATAACG; BRIP Exon7 Rev 5′ -> 3′: GCAGTTAATTTGATTTTCCGAAG; The PCR Mastermix (20 μl/reaction) consisted of 10 μl GoTaq^®^ G2 Hot Start Master Mix (Promega),6 μl nuclease free water; 1 μl forward primer (10 μM); 1 μl reverse primer (10 μM) and 2 μl DNA (15 ng).

### Analysis of TP53 C277Y mutation

Primers for exon 8 were selected according to Abaigar *et al.* 2016 [58]. In brief, the following primer sequences were used: P53 Exon8 For 5′->3′: GGACAGGTAGGACCTGATTTC; TP53 Exon8 Rev 5′->3′: TCTCCATCCAGTGGTTTCTTC; The PCR mastermix (20 µl/reaction) consisted of 10 µl GoTaq^®^ G2 Hot Start Master Mix (Promega); 6 µl nuclease free water; 1 µl forward primer (10 µM); 1 µl reverse primer (10 µM) and 2 µl DNA (15 ng). PCR conditions were again chosen according to Abaigar *et al.* 2016 [58]. The PCR product was purified with ExoSAP-IT^™^ PCR product cleanup reagent. 4 µl ExoSAP-IT^®^ reagent and 10 µl PCR product were mixed and incubated at 37° C for 15 min and then deactivated by incubation at 80° C for 15 min. Sequencing reaction was done with the ABI Prism^®^ BigDye^®^ Terminator v3.1 cycle sequencing chemistry. The sequencing reaction mix (10 µl) consisted of 1 µl BigDye^®^ Terminator, 2 µl BigDye^®^ Terminator v3.1 reaction mix, 4, 5 µl nuclease free water, 0,5µl forward primer (10 µM), 0, 5 µl reverse primer (10 µM) and 2 µl clean PCR product.

### Patient informed consent

Informed consent of the patient for reporting the results was obtained. There are no conflicts of interest for this report for any of the authors.
